# Centripetal filling and pathological insights: a rare case of sporadic renal hemangioblastoma with literature review

**DOI:** 10.3389/fphys.2025.1604834

**Published:** 2025-09-29

**Authors:** Lin Guo, Botao Tang, Sheng Chen, Peng Jiang, Ting Zhang, Taisheng Liang, Jibing Chen, Hongjun Gao

**Affiliations:** ^1^ Graduate School, Guangxi University of Chinese Medicine, Nanning, Guangxi, China; ^2^ Ruikang Hospital, Guangxi University of Chinese Medicine, Nanning, Guangxi, China

**Keywords:** hemangioblastoma, centripetal filling, pathological, review, renal hemangioblastoma

## Abstract

**Background:**

Renal hemangioblastoma (RH) is an uncommon benign tumor primarily found in the central nervous system (CNS), with an exceptionally rare occurrence in the kidney. Its imaging characteristics closely resemble those of malignant tumors, such as renal cell carcinoma (RCC), and its histological features are similar to other hypervascular tumors, including RCC and angiomyolipoma (AML). Consequently, diagnosing RH presents significant challenges. To date, only approximately 31 cases of RH have been reported worldwide, most of which are not associated with Von Hippel-Lindau (VHL) disease. This article presents a case of sporadic RH, supplemented by a comprehensive literature review, with the aim of enhancing the understanding of this condition. The paper will explore its imaging and pathological characteristics, discuss its clinical significance for diagnosis and management, and provide clinicians with valuable insights for differential diagnosis and treatment strategies.

**Case presentation:**

A 48-year-old male patient was admitted after a routine physical examination revealed a mass in his left kidney. Abdominal computed tomography (CT) showed a solid mass in the upper pole of the left kidney, measuring approximately 6.9 × 5.7 × 5.6 cm with well-defined borders. Contrast-enhanced imaging demonstrated peripheral enhancement of the mass in a “centripetal filling” pattern. Following consultation, we had ultimately performed a nephron-sparing surgery (NSS). Postoperative pathology confirmed sporadic RH. Immunohistochemistry results showed positivity for S-100, inhibin-α, and Neuron-Specific Enolase (NSE), further supporting the diagnosis. During the 9-month postoperative follow-up period, the patient remained free of clinical recurrence.

**Conclusion:**

This case report and literature review summarize the clinical features, imaging manifestations, and pathological characteristics of RH. Immunohistochemical markers, including Inhibin-α, S-100, and NSE, are essential for the diagnosis of RH. These markers assist in differentiating RH from other renal tumors, such as RCC and AML, which may present with similar histological features. For patients with minimal symptoms, NSS is the preferred treatment option, as it optimizes renal function preservation and avoids unnecessary overtreatment. This article provides valuable insights for clinicians on the differential diagnosis and treatment strategies for RH, highlighting the importance of a comprehensive evaluation that integrates imaging, pathology, and immunohistochemical findings.

## 1 Introduction

Hemangioblastoma (HB) is a rare benign tumor that arises from the proliferation of mesenchymal cells, primarily affecting the central nervous system (CNS), particularly the cerebellum. It is frequently associated with Von Hippel-Lindau disease (VHL) (Nonaka et al., 2007; Ip et al., 2010). To date, approximately 200 cases of extra-axial HB have been reported, involving peripheral nerves, soft tissues, the spinal cord, liver, retroperitoneum, and other sites, with only 31 cases documented in the kidney (Bisceglia et al., 2018). Due to diagnostic challenges, the imaging features of renal hemangioblastoma (RH) can closely resemble those of other hypervascular renal tumors, such as renal cell carcinoma (RCC) or angiomyolipoma (AML). Microscopically, RH is characterized by sheets of oval or polyhedral cells with pale eosinophilic and microvacuolated cytoplasm, separated by a capillary network and interspersed with larger thin-walled cells. These distinctive features are highly suggestive of HB and help differentiate RH from other renal tumors (Wu et al., 2015). The high degree of histological and immunohistochemical similarity between HB and Clear Cell Renal Cell Carcinoma (ccRCC) makes their clinical differentiation challenging (Montironi et al., 2014; Lei et al., 2023). Given that RH is a benign tumor with slow growth and a favorable prognosis, accurate diagnosis is crucial to prevent misdiagnosis and avoid overtreatment (Ferlay et al., 2018). In this article, we present a case of sporadic RH and provide a literature review to offer insights into the clinical differential diagnosis of RH.

## 2 Case report

On 9 October 2023, a 48-year-old male patient was admitted to the hospital after a left renal mass was discovered during a routine physical examination, prompting further evaluation. The patient denied experiencing symptoms such as hematuria, flank or renal region discomfort, significant weight loss, poor appetite, or any neurological signs. Physical examination revealed no abnormalities, and the patient reported no personal or family history of VHL disease or tumors.

### 2.1 Imaging findings

**FIGURE 1:**
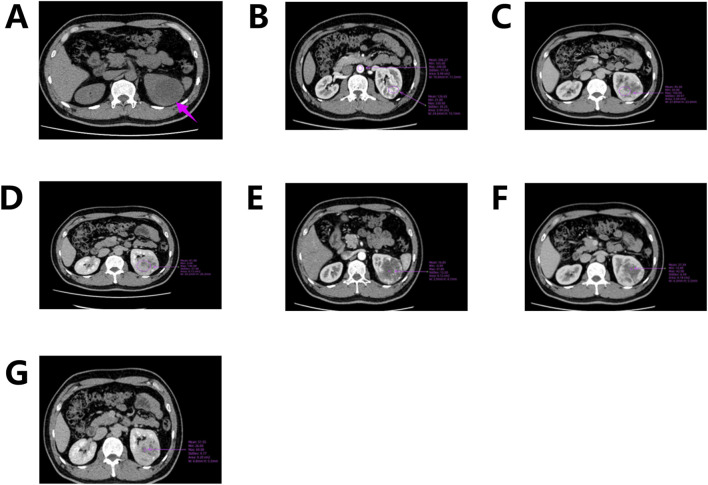
CT scan of the left kidney. **(A)** CT scan shows a mass in the upper pole of the left kidney; **(B)** Corticomedullary Phase; **(C)** Nephrographic Phase; **(D)** Excretory Phase; **(E–G)** Enhanced CT shows a dynamic “centripetal filling” pattern.

Abdominal computed tomography (CT) with contrast enhancement and three-dimensional reconstruction revealed a slightly hypodense lesion with localized calcification in the upper pole of the left renal parenchyma. The lesion measured approximately 6.9 cm × 5.7 cm × 5.6 cm (Figure 1A) and extended beyond the renal contour. The mass was well-circumscribed, roundish, and encapsulated, with no evidence of peripheral invasion. Scattered calcifications were noted. Contrast-enhanced imaging demonstrated predominantly marginal enhancement with a “centripetal filling” pattern, persistent enhancement during the medullary phase, and heterogeneous enhancement during the cortical phase, with the lesion appearing less dense than the adjacent renal parenchyma (Figures 1B–G). “Centripetal filling” is defined as a dynamic contrast enhancement pattern in which contrast agent gradually spreads from the periphery of the tumor toward the center in multiple CT scans.

The right kidney showed no significant abnormalities in position, size, or morphology, and no focal density abnormalities or abnormal enhancement were observed. The bilateral renal pelvises, calyces, and ureters were not dilated. The bladder was adequately filled, with no abnormalities detected. No significant lymphadenopathy was noted in the abdomen or retroperitoneum. Although RCC was highly suspected, the patient’s blood and urine routine tests were normal, and serum tumor markers showed no abnormalities. Furthermore, imaging indicated that the mass was encapsulated with no evidence of peripheral invasion. Considering the patient’s clinical history, we determined that a radical nephrectomy could lead to excessive loss of renal tissue. As a result, a partial nephrectomy was performed.

### 2.2 Postoperative pathological findings

**FIGURE 2:**
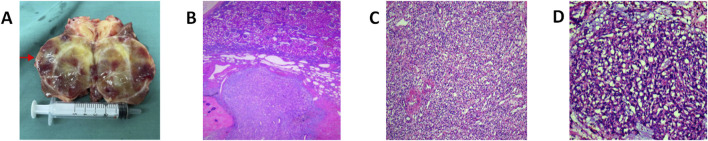
Macroscopic and microscopic features of RH: **(A)** The tumor was seen as a solitary, well-defined mass with intact peritoneum, clearly demarcated from the surrounding renal tissues by the naked eye, and the section was grayish-reddish-grayish-white in color. **(B)** HEx40: microscopically, the tumor is clearly demarcated from the surrounding tissues, and thick fibrous peritumor is seen in some areas; the mesenchymal stroma of the tumor is widely edematous and mucoid, with fibrosis and focal chondroplasia and calcification, and more dilated thick-walled blood vessels are seen in the peri-tumor area. **(C)** HEx100: abundant large polygonal mesenchymal stromal cells with abundant cytoplasm, eosinophilic, diverse cell morphology, different sizes of nuclei,and absence of obvious nuclear atypia are seen between the slender capillary networks. **(D)** HEx200: the tumor is composed of a rich capillary network, with abundant cytoplasmic, pale eosinophilic or hyaline mesenchymal stromal cells interspersed between the vascular network, with a mild cellular morphology, and some cytoplasmic vacuoles of varying sizes; no obvious nuclear atypia is observed.

Macroscopically, the tumor appeared as a solitary, well-demarcated, solid mass with an intact capsule, clearly separated from the surrounding renal tissue. The cut surface exhibited a grayish-red and grayish-white appearance (Figure 2A). Microscopically, the tumor was well-demarcated from the surrounding tissue, with a thick fibrous capsule evident in some areas. The tumor consisted of a rich capillary network interspersed with stromal cells that had abundant, lightly eosinophilic, or clear cytoplasm. These cells showed mild cytological atypia, with some containing vacuoles of varying sizes, and mitotic figures were rare. The tumor stroma displayed extensive edema, myxoid changes, fibrosis, and focal chondroid metaplasia and calcification. Dilated, thick-walled vessels were observed surrounding the tumor (Figures 2B–D).

### 2.3 Immunohistochemical results

**FIGURE 3:**
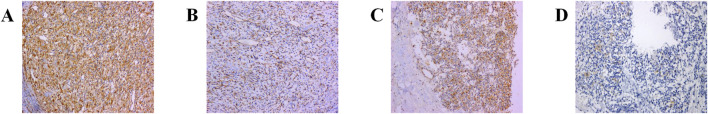
Immunohistochemical manifestations of primary RH: **(A)** Vimentin (+): strong positivity in the tumor interstitium (confirming mesenchymal origin). **(B)** CD31 (+): CD31-positive vascular network was seen in the tumor interstitium, and numerous capillaries were seen in the tumor (highlighting characteristic capillary-rich architecture). **(C)** a-inhibin (+): immunohistochemical positivity for markers such as inhibin in tumor and stromal cells consisting of large, multivacuolated and adipose stromal cells as well as abundant capillary networks (key marker differentiating from renal cell carcinoma). **(D)** NSE exhibits diffuse and strong reactivity (supporting neuroendocrine differentiation). Note: This immunoprofile (Inhibin-α+/S-100+/PAX8-) is distinct from ccRCC or AML.

Immunohistochemical results showed that cytokeratin-P (CK-P) was negative, smooth muscle actin (SMA) was negative, epithelial membrane antigen (EMA) was negative, Vimentin was strongly positive in stromal cells, S-100 was partially positive in stromal cells, HMB45 was negative, CD34 was positive in vascular endothelial cells, and Ki-67 was approximately 2% positive. Additional immunohistochemical staining revealed: Vimentin was positive, PAX-8 was weakly positive, D2-40 was negative, Calretinin (CR) was negative, Melan-A was negative, and Ki-67 remained approximately 2% positive. Stromal cells were positive for Inhibin-α and Neuron-Specific Enolase (NSE) was weakly positive, while vascular endothelial cells were positive for CD31 and ERG (Figures 3A–D).

## 3 Discussion

The R.E.N.A.L. and PADUA scoring systems are surgical tools used to assess the anatomical characteristics of renal tumors, and they are currently the most widely applied in clinical practice. In this case, the tumor scored 10 points on the R.E.N.A.L. system, indicating high complexity (maximum tumor diameter between 4 and 7 cm, 2 points; exophytic/endophytic proportion <50%, 2 points; distance to the collecting system ≤4 cm, 3 points; tumor crossing the polar line and midline, 3 points). Similarly, the PADUA scoring system assigned a score of 12 points, also classifying the tumor as highly complex (longitudinal location—upper or lower pole, 1 point; medial/lateral position—neither, 3 points; relationship to renal sinus—involved, 2 points; relationship to collecting system—involved, 2 points; exophytic rate—<50%, 2 points; maximum tumor diameter—4.1–7 cm, 2 points).

Due to the limited understanding of RH, it is often misdiagnosed, particularly in cases like this one, where the tumor is endophytic, large in diameter, and closely associated with the renal sinus and collecting system. Misdiagnosis frequently leads to radical nephrectomy, which is unnecessary since RH is a benign renal tumor, and nephron-sparing surgery (NSS) is the optimal treatment approach. The successful implementation of NSS for highly complex RH cases heavily depends on the surgeon’s meticulous technique, with careful attention to avoiding adverse outcomes such as positive surgical margins or intraoperative bleeding. The surgeon in this case, with extensive clinical experience, thoroughly considered the patient’s medical history, biological characteristics, and anatomical features, all of which supported the decision to perform a partial nephrectomy. Accurate diagnosis is crucial to prevent overtreatment. As a result, we reviewed cases of RH to identify key diagnostic features.

### 3.1 Is VHL disease related to RH?

VHL disease is associated with mutations in the VHL tumor suppressor gene located on chromosome 3p, and HB is considered a hallmark feature of this condition. The clinical diagnosis of VHL disease is based on the presence of HBs in the CNS or retina, the existence of a typical VHL-associated tumor (such as ccRCC, pheochromocytoma, neuroendocrine tumors, etc.), or a family history of the disease. If these criteria are not met, the condition is classified as sporadic (Karabagli et al., 2007). As such, the patient in this case was diagnosed with sporadic RH.

Regarding the pathogenesis of VHL, Knudson et al. proposed that the development of VHL-associated tumors results from a germline monoallelic defect in the patient, combined with somatic mutations induced by other factors (Knudson, 1991; Kondo and Kaelin, 2001). Current clinical genetic testing methods for detecting germline mutations in families with VHL disease have an accuracy rate of nearly 100%. Therefore, genetic testing is strongly recommended for patients diagnosed with HB, as it plays a crucial role in prognosis, treatment planning, and may also provide valuable information for the patient’s immediate family members (Bisceglia et al., 2018).

A study reported chromosomal abnormalities in 10 cases of sporadic cerebellar HB without VHL disease using comparative genomic hybridization. The abnormalities included losses in chromosomes 3 (70%), 6 (50%), 9 (30%), and 18q (30%), as well as gains in chromosome 19 (30%) (Sprenger et al., 2001). Another study observed chromosomal abnormalities in 7 out of 22 cases of HBs, both with and without VHL (Lemeta et al., 2002). However, in existing reports on RH, no significant abnormalities were detected in 10 tumor patients using polymerase chain reaction for VHL gene exons, loss of heterozygosity on chromosome 3p, fluorescence *in situ* hybridization for chromosome 3p deletion, or next-generation sequencing (Wang et al., 2022). Furthermore, patients who did not undergo genetic testing were not reported to have a family history of VHL or related clinical symptoms (details in Table 1). Therefore, we conclude that there is currently insufficient evidence to support the hypothesis that RH is associated with VHL.

**TABLE 1:** Patient characteristics and family history of VHL-Related conditions.

Author and year	Sex (M/F)	Age	Size (cm)	Treatment	Clinical symptom	Location of disease	Presence of VHL disease
Nonaka et al., 2007	F	71	6.8 × 6.0 × 2.5	radical nephrectomy	n.i	upper right	n.i
Ip et al., 2010	M	58	5.5	n.i	hematuria	right center	untested
	F	55	3.5	n.i	abdominal pain	right	untested
Verine et al., 2011	M	64	3.2	partial nephrectomy	asymptomatic	upper left	untested
Liu et al., 2012	F	16	1.2	radical nephrectomy	hematuria	upper left	untested
Wang et al., 2022	M	29	3.1 cm	radical nephrectomy	asymptomatic	lower right side	untested
Yin et al., 2012	M	61	5.3 × 5.0 × 5.0	radical nephrectomy	asymptomatic	upper right	untested
Jiang et al., 2013	F	57	3 × 2	radical nephrectomy	asymptomatic	upper right	untested
Wang et al., 2019	M	61	6.5 × 6.2	radical nephrectomy	asymptomatic	upper right	untested
Zhao et al., 2013	F	51	5.5 × 4.5	radical nephrectomy	abdominal pain	lower right side	untested
Doyle and Fletcher, 2014	M	58	4.5	n.i	Fever/weight loss	right	untested
	F	42	15, 4,2 (3 tumors)	n.i	hematuria	left	untested
	M	29	2.7	n.i	asymptomatic	right	untested
Kurado et al., 2015	M	37	3.5	radical nephrectomy	asymptomatic	upper left	untested
Wu et al., 2015	M	48	2.3	radical nephrectomy	asymptomatic	right	untested
	M	25	3.6	radical nephrectomy	asymptomatic	left	untested
	F	36	n.i	n.i	asymptomatic	left	untested
	F	30	3.2 × 2.5 × 1.4	n.i	asymptomatic	right	untested
Xu et al., 2017	M	61	3 × 2	radical nephrectomy	n.i	upper left	untested
Muscarella et al., 2018	M	21	3.5	n.i	hematuria	left	untested
	F	19	3	n.i	hematuria	right	untested
	M	47	n.i	n.i	n.i	right	untested
Oberhammer et al., 2019	F	72	4.2 × 3.6 × 4.3	partial nephrectomy	n.i	lower left	untested
He et al., 2021	M	45	3.7 × 4.2	partial nephrectomy	abdominal pain	lower left	untested
	F	42	2.9 × 2.6 × 2.8	partial nephrectomy	abdominal pain	left	untested
Wang et al., 2022	M	61	3.5	partial nephrectomy	hematuria	lower left	untested
Xu et al., 2017	M	40	3 × 3 × 3	radical nephrectomy	asymptomatic	right middle lateral	untested
	F	45	3	radical nephrectomy	asymptomatic	Right center	untested
	M	56	8	radical nephrectomy	asymptomatic	right	untested
Raja et al., 2023	M	n.i	2.4	radiofrequency ablation	asymptomatic	upper left	untested
Lee et al., 2024	M	19	4.1 × 3.5 × 2.8	partial nephrectomy	abdominal pain	upper left	untested
this column	M	48	6.9 × 5.7 × 5.6	partial nephrectomy	asymptomatic	upper left	untested

### 3.2 The role of imaging in the diagnosis of RH

Imaging plays an increasingly important role in diagnosing renal masses and monitoring therapeutic outcomes. CT is the preferred modality for evaluating renal tumors, while magnetic resonance imaging (MRI) and ultrasonography are typically reserved for patients with renal insufficiency or contrast sensitivity. Additionally, percutaneous biopsy can be considered part of the imaging-based evaluation for renal masses, particularly in cases with uncertain etiology, as it provides additional diagnostic information.

In this case, CT revealed a heterogeneously enhanced, roundish solid mass with scattered calcifications, predominantly located along its periphery. While Nonaka et al. previously described calcifications localized within cystic spaces, the calcifications in our case were primarily concentrated at the tumor margins (Nonaka et al., 2007). Contrast-enhanced imaging demonstrated marked enhancement of the peripheral solid component (maximum CT value ∼230 HU; abdominal aorta CT value ∼248 HU), with cortical phase enhancement approaching aortic CT values (Figure 1B). During the medullary phase (CT value ∼160 HU) and excretory phase (CT value ∼146 HU), enhancement diminished slightly (Figures 1C–D). The central hypodense area on non-contrast scans exhibited mild delayed enhancement during cortical, medullary, and excretory phases (CT values ∼37, 42, and 69 HU, respectively), demonstrating progressive “centripetal filling” (Figures 1E–G).

Based on existing literature, the imaging characteristics of RH can be summarized as follows: 1. RH shows no significant sex or renal laterality predilection and predominantly occurs at the upper or lower poles of the kidney (see Table 1) (Nonaka et al., 2007). 2. RH is a benign tumor, typically well-encapsulated, roundish, and sharply marginated. Larger tumors may protrude outward or compress the renal pelvis/calyces inward but lack invasion or metastatic features (He et al., 2021). 3. On CT, RH may present with hyper-, hypo-, or isodense internal components. Some tumors may exhibit cystic degeneration, necrosis, calcifications, or stromal edema. 4. MRI reports on RH remain limited. Current studies suggest T2-weighted hyperintensity resembling hepatic cavernous hemangiomas. Although MRI is a key tool for diagnosing HBs in other locations (e.g., the spine), its application in RH remains underutilized (Capitanio and Montorsi, 2016). He et al. observed significant peripheral enhancement during the cortical phase, “progressive persistent enhancement” during medullary and excretory phases, and a “centripetal filling pattern,” occasionally progressing to complete filling (He et al., 2021).

**FIGURE 4:**
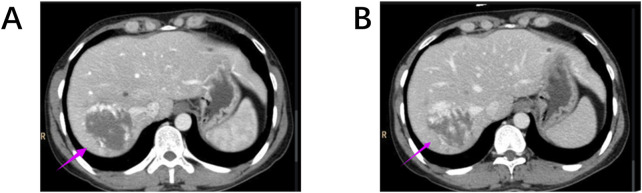
**(A, B)** Hepatic cavernous hemangiomas also exhibit a “centripetal filling” pattern similar to RH on contrast-enhanced CT scans.

This enhancement pattern has not been reported in other renal tumors and aligns with our findings, suggesting it may be a distinctive imaging feature of RH. The “centripetal filling” phenomenon may be attributed to the tumor’s rich capillary network, which leads to cortical-phase CT values comparable to those of the adjacent aorta. Dynamic or multiphase scans reveal enhancement patterns resembling cavernous hemangiomas, likely due to slow intratumoral blood flow and prolonged contrast retention (Figures 4A,B). The heterogeneous enhancement could be related to recurrent intratumoral hemorrhage and fibrosis. However, due to the limited number of detailed imaging reports on RH, larger multicenter studies are required to validate these findings.

Clear cell and papillary RCCs account for 90% of solid renal malignancies, with ccRCC representing approximately 70% (Srigley et al., 2013). Malignant renal tumors are often accompanied by clinical signs such as flank pain, hematuria, lymph node or distant metastases, and renal pelvic or calyceal invasion, which help differentiate them from RH. CCRCC typically appears on CT and MRI as a solitary, roundish, solid or cystic intrarenal mass, occasionally lobulated, and often containing calcifications. Contrast-enhanced imaging reveals marked heterogeneous enhancement with prominent arterial phase enhancement. Although imaging reports for RH are limited, some features of RCC overlap with those of RH, complicating the differentiation between the two.

**FIGURE 5:**
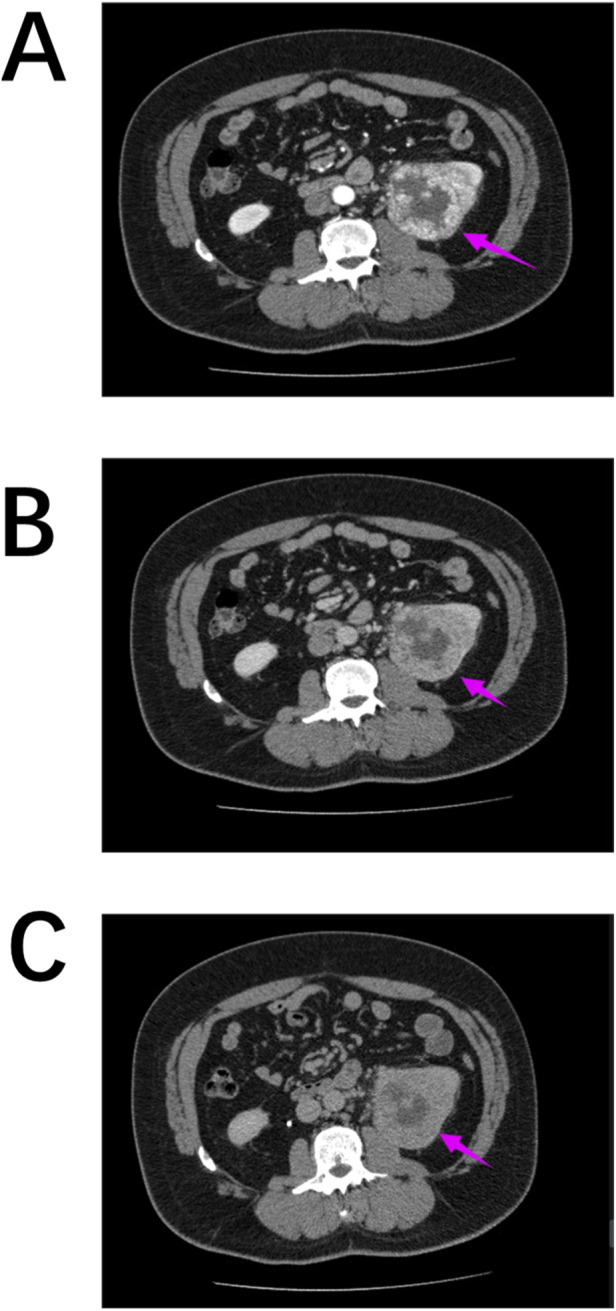
**(A–C)** The distinctive “wash-in, wash-out” pattern of renal clear cell carcinoma on CT scans: **(A)** Corticomedullary Phase; **(B)** Nephrographic Phase; **(C)** Excretory Phase.

A key distinction is the “wash-in, wash-out” enhancement pattern observed in ccRCC (Figures 5A–C). On MRI, ccRCC typically exhibits iso-/hypointense T1 signals, hyperintense or mixed T2 signals, and a hypointense pseudocapsule (Nazari et al., 2020; Ishigami et al., 2014). Papillary RCC often presents as a homogeneous solid mass with well-defined margins, distorting renal contours. It generally shows low vascular density, frequent cystic degeneration or hemorrhage, and rare calcifications. Unenhanced imaging may mimic benign lesions, and some cases demonstrate minimal CT enhancement but marked MRI enhancement, with hypointense T2-weighted signals (Mendhiratta et al., 2021; Couvidat et al., 2014; Walker et al., 2019).

Contrast-enhanced ultrasonography (CEUS) is widely used for renal mass characterization. CCRCC usually displays early hyperenhancement, late washout, pseudocapsule presence, and heterogeneous enhancement that intensifies with tumor size. Papillary RCC often shows slow wash-in, rapid wash-out, homogeneous hypoenhancement, and visible pseudocapsules (Wei et al., 2019; Xue et al., 2015; Gulati et al., 2015). In addition, PET/CT, with its high sensitivity and precise localization, is increasingly used for disease imaging based on molecular and metabolic tumor characteristics. While reported in VHL disease-associated RCC, ccRCC, pheochromocytoma, and multiorgan involvement, no studies have yet explored its utility in RH (Oh et al., 2012; Rizzo et al., 2023; Kaji et al., 2007).

### 3.3 The role of pathology in RH

Renal biopsy is a crucial tool for diagnosing focal renal lesions and guiding treatment strategies. It exhibits high sensitivity (97.5%) and specificity (96.2%) for diagnosing malignancies, with a diagnostic accuracy exceeding 90%, showing strong concordance with nephrectomy results. However, statistics indicate that up to one-fifth of renal masses smaller than 4 cm resected by nephrectomy are benign. The underutilization of renal biopsy contributes to approximately 6,000 unnecessary nephrectomies annually (Campbell et al., 2017; Lim et al., 2019; Patel et al., 2018).

According to existing case reports of RH, including this case, a total of 32 cases have been documented. Of these, 8 patients underwent partial nephrectomy, 13 received radical nephrectomy, 1 underwent radiofrequency ablation, and the remaining cases lacked specified treatment details. Notably, only 1 patient underwent renal biopsy (see Table 1).

At high magnification, RH typically shows a well-defined border with an abundant capillary network and stromal cells, which may exhibit marked pleomorphism. Caution is necessary when differentiating RH from RCC with a distinct vascular system, AML, adrenocortical carcinoma, and pheochromocytoma. CCRCC of the kidney is a key factor in the survival of patients with VHL disease and is a malignant tumor in its own right (Maher et al., 2011; Zhou et al., 2019).

Macroscopically, ccRCC typically exhibits a golden-yellow hue due to its high lipid content. Microscopically, it is characterized by a distinctive microvascular network and nests of neoplastic cells with clear cytoplasm. The recent reporting of ccRCC cases displaying RH-like features has introduced a new challenge in differentiating these two entities (Lei et al., 2023; Setia et al., 2020). Some scholars have raised concerns about whether RH represents a true RH distinct from ccRCC, or if it merely reflects a spectrum of diffuse hemangioblastoma-like differentiation within ccRCC (Montironi et al., 2014).

Immunohistochemistry plays a crucial role in resolving diagnostic challenges. Inhibin-α, S100, and NSE are reliable markers for confirming RH, while PAX8, PAX2, CD10, and EMA are typically consistently positive in ccRCC. However, there have been reports of RH cases exhibiting positivity for PAX8, PAX2, CD10, and EMA. Additionally, scholars suggest that hemangioblastoma-like components in ccRCC may also express inhibin-α. Therefore, when the diagnosis is challenging based on morphology and the initial immunoprofile, additional immunohistochemical staining for pan-cytokeratin, S100, NSE, and inhibin-α is essential (Zhao et al., 2013; Yin et al., 2012).

PAX8 and PAX2 are cell lineage-specific transcription factors involved in the regulation of important molecular pathways in kidney development and are expressed in many renal diseases such as RCC (Wang et al., 2022; Chi and Epstein, 2002). CD10, on the other hand, is highly suggestive of a proximal tubular cell origin and is considered a marker for clear cell and papillary RCC (Yin et al., 2012). Thus, Zhao et al. proposed that differences in the immunophenotype of HBs outside the CNS correlate with differences in site of origin (Zhao et al., 2013; Yin et al., 2012). Research indicates that CNS HBs exhibit consistent expression of glucose transporter 1 (GLUT1) in their vascular endothelial cells, mirroring the GLUT1 expression pattern observed in normal CNS vasculature. In contrast, RHs demonstrate a lack of GLUT1 expression in their endothelial cells, aligning instead with the GLUT1-negative profile characteristic of normal peripheral blood vessels (Yin et al., 2012; Lee et al., 2024).

Therefore, based on the available reports, we believe that the appearance of changes in the RH immune profile is associated with heterogeneous origins or differentiation. Microscopically, epithelioid AML may also exhibit large, thick-walled vessels and polygonal cells. However, the presence of “spidery” cytoplasm and frequent perivascular hyalinization may help differentiate it from RH. Additionally, HMB45 and MelanA are usually positive in AML, which can further aid in distinguishing it from RH (Nese et al., 2011).

Patients with RH often present clinically as asymptomatic or with abdominal pain and hematuria. In such cases, adrenocortical carcinoma and the rarer pheochromocytoma can be distinguished by their more specific clinical manifestations. Adrenocortical carcinoma often presents with signs and symptoms of hormone overdose, while pheochromocytoma is characterized by persistent or paroxysmal hypertension, often accompanied by headaches and palpitations. These conditions can be differentiated from RH in conjunction with other laboratory tests (Else et al., 2014). Additionally, there have been several reports of cases in which rhabdomyosiform features have been observed microscopically. This necessitates a strict differentiation between RH and other tumors with rhabdomyosiform features, such as RCC with rhabdomyosiform features, malignant rhabdomyomas (e.g., AML, HMB45+ and MelanA+), ccRCC with rhabdomyosiform features (PAX8+, PAX2+, and CD10^+^), and pheochromocytomas (synaptophysin+, chromogranin+, and α-inhibin+), among others (Oberhammer et al., 2019; Xu et al., 2017). Since different tumors can present with multiple histological manifestations due to morphological variability, it is essential to accumulate comprehensive RH case reports. Accurate histologic/pathologic results are crucial for improving the accuracy of renal biopsy, standardizing the process of RH diagnosis and treatment, and reducing overtreatment (Table 2).

**TABLE 2:** Comparison between RH and ccRCC, AML.

Feature		Renal hemangioblastoma (RH)	Clear cell renal cell Carcinoma (CCRCC)	Angiomyolipoma (AML)
Imaging Findings	CT scan	Equal or slightly lower density, with clear boundaries	Equal or slightly lower density, unclear boundaries	Uneven density (containing fatty components, CT value < −20 HU)
	CT enhancement	“centripetal filling”	“wash-in, wash-out”	uneven strengthening
	MRI	Equal to or slightly lower signal	Equal to or slightly lower signal	High signal (fat content), other components, *etc.*,/low signal
Pathology	General appearance	Clear boundaries, intact capsule, grayish red or grayish white cross section	Golden yellow (rich in lipids), unclear boundaries, visible necrosis/hemorrhage	Yellow to grayish red, soft texture, clear boundaries, containing fat, blood vessels, and smooth muscle
	Microscopy	Interstitial cells + rich capillary network (lightly stained cytoplasm or vacuolar)	Transparent cells + rich vascular network (transparent cytoplasm)	Mixed components: fat, blood vessels, smooth muscle (spindle cells)
	IHC Markers	Inhibin-α(+), S-100 (+), NSE(+); CK(−), EMA (−)	PAX8 (+), CD10 (+), EMA (+); Inhibin-α(−)	HMB45(+), Melan-A (+), SMA (+); CK(−)
	clinical symptoms	Incidental (asymptomatic), rare hematuria/flank pain	Hematuria, flank pain, palpable mass, weight loss (advanced)	Asymptomatic (if small); spontaneous bleeding/rupture (if large)
	Syndromes	All reported cases to date have been sporadic	Sporadic or VHL-related (10%)	Sporadic or tuberous sclerosis complex -associated (20%)
	Treatment	Nephron-sparing surgery (standard)	Radical/partial nephrectomy (stage-dependent)	Surgery/embolization (if > 4 cm or symptomatic)
	prognosis	Benign	Malignant (5-year survival: Stage I >90%, Stage IV <10%)	Benign (rupture risk size-dependent)

## 4 Limitations and future research

This report is based on a single case of sporadic RH, and due to the rarity of RH and the absence of large cohort studies, the imaging and pathological findings presented in this case may not be universally applicable. While our findings contribute to advancing the understanding of RH, it is important to note that the conclusions drawn may not apply to all cases of RH due to the limited number of reported cases and the lack of large-scale data. Future multicenter studies or comprehensive case registries will be essential for validating the imaging and pathological features observed in this case. These larger datasets will enable the establishment of more robust and generalizable conclusions, which could ultimately improve clinical practice and diagnostic accuracy.

## 5 Conclusion

RH is a benign yet poorly understood renal tumor. Clinical management should prioritize integrating patient history, imaging, and biopsy for indeterminate solid masses. Active surveillance is appropriate for non-surgical cases, while NSS performed by experienced teams optimizes outcomes in operable scenarios. Accumulating case data and systematic research are crucial to better understanding RH’s biological behavior, refining diagnostic criteria, and exploring potential associations with VHL disease. These advancements will help bridge current knowledge gaps and lead to more precise therapeutic strategies.

## Abbreviations

RH, Renal hemangioblastoma; RCC, renal cell carcinoma; VHL, Von Hippel-Lindau; HB, Hemangioblastoma; CT, Abdominal computed tomography; NSE, Neuron-Specific Enolase; AML, Angiomyolipoma; CNS, central nervous system; ccRCC, Clear Cell Renal Cell Carcinoma; CK-P, cytokeratin-P; SMA, smooth muscle actin; EMA, epithelial membrane antigen; CR, Calretinin; NSS, nephron-sparing surgery; MRI, magnetic resonance imaging; CEUS, Contrast-enhanced ultrasonography; GLUT1, glucose transporter 1.
